# Selection of Appropriate Metagenome Taxonomic Classifiers for Ancient Microbiome Research

**DOI:** 10.1128/mSystems.00080-18

**Published:** 2018-07-17

**Authors:** Irina M. Velsko, Laurent A. F. Frantz, Alexander Herbig, Greger Larson, Christina Warinner

**Affiliations:** a Palaeogenomics and Bio-Archaeology Research Network, Research Laboratory for Archaeology and the History of Art, University of Oxford, Oxford, United Kingdom; b School of Biological and Chemical Sciences, Queen Mary University of London, London, United Kingdom; c Department of Archaeogenetics, Max Planck Institute for the Science of Human History, Jena, Germany; d Department of Anthropology, University of Oklahoma, Norman, Oklahoma, USA; e Department of Periodontics, University of Oklahoma Health Sciences Center, Oklahoma City, Oklahoma, USA; Oregon State University

**Keywords:** ancient DNA, metagenomics, microbiome, taxonomy

## Abstract

Metagenomics enables the study of complex microbial communities from myriad sources, including the remains of oral and gut microbiota preserved in archaeological dental calculus and paleofeces, respectively. While accurate taxonomic assignment is essential to this process, DNA damage characteristic of ancient samples (e.g., reduction in fragment size and cytosine deamination) may reduce the accuracy of read taxonomic assignment. Using a set of *in silico*-generated metagenomic data sets, we investigated how the addition of ancient DNA (aDNA) damage patterns influences microbial taxonomic assignment by five widely used profilers: QIIME/UCLUST, MetaPhlAn2, MIDAS, CLARK-S, and MALT. *In silico*-generated data sets were designed to mimic dental plaque, consisting of 40, 100, and 200 microbial species/strains, both with and without simulated aDNA damage patterns. Following taxonomic assignment, the profiles were evaluated for species presence/absence, relative abundance, alpha diversity, beta diversity, and specific taxonomic assignment biases. Unifrac metrics indicated that both MIDAS and MetaPhlAn2 reconstructed the most accurate community structure. QIIME/UCLUST, CLARK-S, and MALT had the highest number of inaccurate taxonomic assignments; false-positive rates were highest by CLARK-S and QIIME/UCLUST. Filtering out species present at <0.1% abundance greatly increased the accuracy of CLARK-S and MALT. All programs except CLARK-S failed to detect some species from the input file that were in their databases. The addition of ancient DNA damage resulted in minimal differences in species detection and relative abundance between simulated ancient and modern data sets for most programs. Overall, taxonomic profiling biases are program specific rather than damage dependent, and the choice of taxonomic classification program should be tailored to specific research questions.

**IMPORTANCE** Ancient biomolecules from oral and gut microbiome samples have been shown to be preserved in the archaeological record. Studying ancient microbiome communities using metagenomic techniques offers a unique opportunity to reconstruct the evolutionary trajectories of microbial communities through time. DNA accumulates specific damage over time, which could potentially affect taxonomic classification and our ability to accurately reconstruct community assemblages. It is therefore necessary to assess whether ancient DNA (aDNA) damage patterns affect metagenomic taxonomic profiling. Here, we assessed biases in community structure, diversity, species detection, and relative abundance estimates by five popular metagenomic taxonomic classification programs using *in silico*-generated data sets with and without aDNA damage. Damage patterns had minimal impact on the taxonomic profiles produced by each program, while false-positive rates and biases were intrinsic to each program. Therefore, the most appropriate classification program is one that minimizes the biases related to the questions being addressed.

## Abstract (precis)

Ancient biomolecules from oral and gut microbiome samples have been shown to be preserved in the archaeological record. Studying ancient microbiome communities using metagenomic techniques offers a unique opportunity to reconstruct the evolutionary trajectories of microbial communities through time. DNA accumulates specific damage over time, which could potentially affect taxonomic classification and our ability to accurately reconstruct community assemblages. It is therefore necessary to assess whether ancient DNA (aDNA) damage patterns affect metagenomic taxonomic profiling. Here, we assessed biases in community structure, diversity, species detection, and relative abundance estimates by five popular metagenomic taxonomic classification programs using *in silico*-generated data sets with and without aDNA damage. Damage patterns had minimal impact on the taxonomic profiles produced by each program, while false-positive rates and biases were intrinsic to each program. Therefore, the most appropriate classification program is one that minimizes the biases related to the questions being addressed.

## INTRODUCTION

Ancient microbiome research offers the possibility of tracing the evolution of the complex microbial communities that play an integral role in shaping population health and disease. Paleomicrobiology uses archaeological material to trace the emergence and spread of microorganisms throughout history and prehistory. Archaeological dental calculus and paleofeces are promising substrates for ancient human microbiome studies, since they have been shown to preserve DNA (1), proteins (1, 2), and small-molecule metabolites (3) from both resident microbes and the host. During life, these dense microbial communities contain hundreds of species, predominantly composed of bacteria (4), but also including archaea (4), viruses (5), fungi (6), and protists (7). Characterization of the microbial ecology of host-associated microbiota through time is a necessary step in understanding the function of these microbial communities and in assessing how they interact with the host.

DNA derived from archaeological samples, including ancient microbial samples, is known to acquire predictable damage patterns, including short fragment lengths (typically <100 bp) (8) with breakpoints primarily coinciding with depurination and accumulation of cytosine-to-thymine transitions at the ends of the molecules (8). The ubiquity and predictability of these damage patterns mean that they are often used to authenticate ancient DNA and estimate modern DNA contamination (9, 10), and the short fragment lengths of ancient DNA negate the need for shearing during library construction for high-throughput sequencing (HTS). The same properties, however, potentially affect taxonomic classification of microbial DNA sequencing reads, making them more difficult or less accurate. Reads that are too short, for example, may not be specific enough for classification at the taxonomic level desired. Cytosine-to-thymine transitions may also cause misclassification or prevent classification, such that reads may be misleadingly assigned to unidentified taxa, thereby inflating diversity estimates. Additionally, although 16S rRNA gene amplicon sequencing is popular for profiling complex microbial communities, taxon-specific length polymorphisms in this gene combined with the relatively long lengths of the hypervariable regions (>150 bp) make it difficult to avoid amplification biases and taxonomic dropout when reconstructing microbial community profiles from degraded ancient (aDNA) (8). Instead, shotgun metagenomic sequencing, which is highly compatible with short DNA fragments, is the preferred analytical approach for ancient microbiome samples (1, 11).

Community profiling by DNA shotgun sequencing is currently the most comprehensive method used to assess microbiome community composition, and a variety of computational tools are available to reconstruct the species present from the millions of short sequences that comprise next-generation sequencing (NGS) data sets. There are several methods for taxonomic assignment available. Popular methods include matching reads to 16S rRNA gene sequences (e.g., QIIME [12] and Mothur [13]) or to single-copy gene panels (e.g., MetaPhlAn2 [14, 15], MIDAS [16], and PhyloSift [17]), k-mer-based whole-genome matching (e.g., Kraken [18] and CLARK [19, 20]), and hybrid k-mer-based matching and alignment extension (MALT [21, 22]). While there are several publications comparing the accuracies, specificities, and precisions of various metagenomic classification programs for modern samples (e.g., see references 23–25), no study has yet compared the performance of these approaches on ancient DNA data sets.

In order to assess the performance of metagenomic classification systems on ancient DNA, we performed a comparison of the community profiles of six metagenomic classification programs that use different taxonomic assignment methods (QIIME/UCLUST, MetaPhlAn2, MIDAS, CLARK-S, and MALT in BLAST-X mode). To estimate the accuracy of these programs on known community profiles, we performed our analysis on *in silico*-generated modern and ancient metagenome samples. For the latter data set, DNA damage patterns were modeled after degradation patterns observed in historic dental calculus samples from the Radcliffe Infirmary burial ground (ca. 1770 to 1855; Oxford, England) (3). Our results indicate that the effect of DNA damage patterns on taxonomic assignments is variable across programs, but most of the programs tested here are susceptible to misassignment of only a small proportion of reads due to DNA damage. Overall, our results indicate that for highly abundant species, taxonomic assignment biases are similar between modern and ancient simulated metagenomic data sets.

## RESULTS

We generated 39 *in silico* metagenomic community data sets using independent runs of the aDNA sequence simulator gargammel (26) (see Table S1). Three overlapping sets of 40, 100, and 200 total genomes were used as input. All genomes in the 40-genome set were included in the 100-genome set, and all genomes in the 100-genome set were included in the 200-genome set (see Table S1). Each genome was represented in equal abundance, wherein each genome in the 40-, 100-, and 200-genome data sets comprises 2.5%, 1%, and 0.5% of the total DNA, respectively. There were 13 independent samples for each set of genomes, where 10 replicates had simulated aDNA damage patterns (ancient data set) and three replicates did not have aDNA damage patterns (effectively mimicking a modern data set). The estimated copy number of each genome in each data set is presented in Table S1.

**FIG 1:**
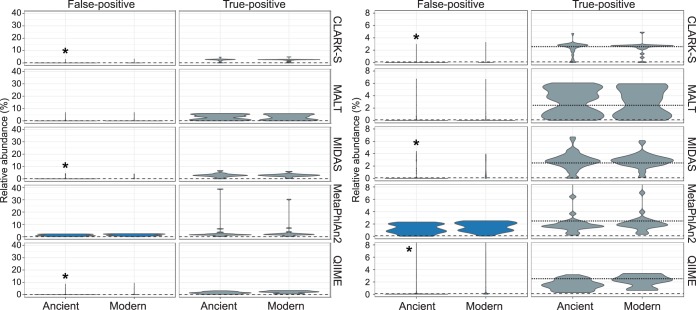
Distribution of false-positive and true-positive assignments in simulated ancient and modern 40 genome samples. The majority of false-positive assignments by CLARK-S, MALT, MIDAS, and QIIME/UCLUST are below the arbitrary cutoff value of 0.1% relative abundance (indicated by the long dotted line). The left set of panels shows the full extent of relative abundance for all assignments. The right set of panels is the same as the left set, but relative abundance is restricted to below 8% to better visualize the distribution of taxa. The short dotted line at 2.5% in the true-positive panel indicates the true relative abundance of DNA from each genome. *, *P* < 0.05.

We filtered the output profiles to remove species present at <0.1% abundance to understand how filtering of low-abundance taxa affected diversity metrics. The cutoff of 0.1% was selected based on reference 23. In general, application of this filtering step reduced the number of false-positive species reported in ancient simulated data sets for CLARK-S, MetaPhlan2, MIDAS, and QIIME/UCLUST (Fig. 1; see Fig. S2A to D). We found the number of false-positive assignments was significantly higher in simulated aDNA libraries than in simulated modern DNA libraries (Fisher’s exact test) for CLARK-S, MetaPhlan2, MIDAS, and QIIME/UCLUST (see Table S4). MALT and MetaPhlan2 were the only two programs that show no significant enrichment of false-positive assignment when using aDNA (see Table S4).

### Community structures are consistent between the ancient and modern simulated data sets.

**FIG 2:**
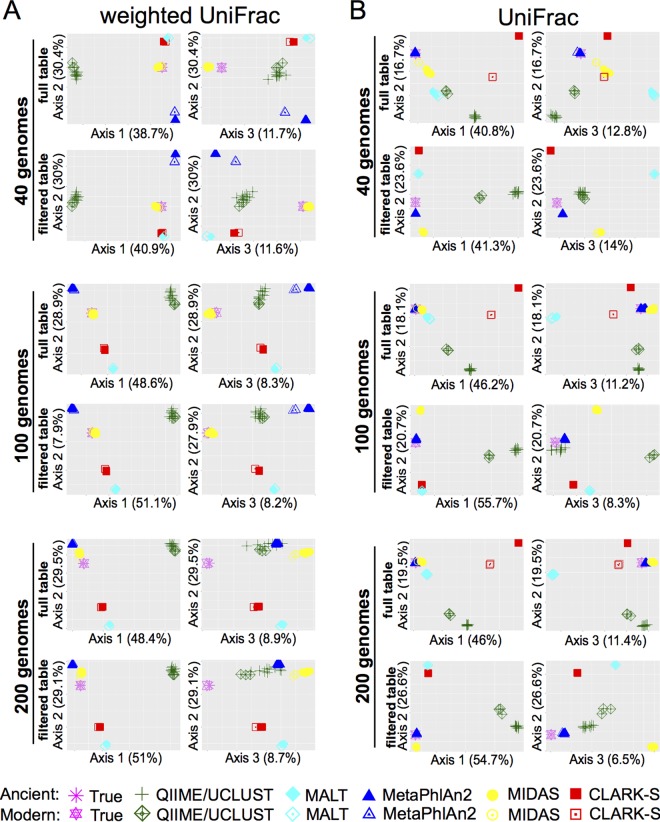
Damage patterns minimally influence reported phylogenetic-based community structure. (A) Principal-coordinate analysis plots of abundance-weighted UniFrac beta diversity for data sets made with 40, 100, and 200 genomes for full-output tables and tables filtered to remove species present at <0.1% abundance. (B) Principal-coordinate analysis plots of UniFrac beta diversity for data sets made with 40, 100, and 200 genomes for full-output tables and tables filtered to remove species present at <0.1% abundance.

We first sought to determine if any of the taxonomic classification programs produced a community structure that closely resembled the true input files by measuring beta diversity. We used both weighted UniFrac (phylogenetic relatedness accounting for relative abundance of organisms) and unweighted UniFrac (phylogenetic relatedness without accounting for relative abundance of organisms) metrics on full and filtered (>0.1% abundance) tables. Principal-coordinate analyses (PCoAs) of the beta-diversity metrics were plotted to visualize relatedness between community structure of the input files and community structure as determined by the five successfully tested programs (Fig. 2A and B). This analysis demonstrated that classification of replicate samples was highly consistent within each program, although QIIME showed the greatest variance between replicates. Filtering out low-abundance species did not affect the weighted UniFrac distance, as this metric accounts for relative abundance of species, and therefore removing low-abundance species minimally affects the final score. Additionally, there were relatively small differences in the scores of the ancient and modern data sets for all programs, although QIIME/UCLUST demonstrated the greatest age-related difference in beta diversity. MIDAS-determined community structure calculated by weighted UniFrac distance was most similar to the input files for 40- and 100-genome data sets (Fig. 2A). CLARK-S and MALT community structures were more similar to each other than to any of the other programs for all data sets, while the community structures reconstructed using QIIME/UCLUST and MetaPhlAn2 were each distinct from the other programs and did not plot near any other programs in the PCoA (Fig. 2A). Using the nonphylogenetic abundance-weighted Bray-Curtis distance, we observed similar PCoA plotting patterns by each group, relative to the true input, at the species and genus levels (see Fig. S3 to S5).

Plots of beta diversity by the unweighted UniFrac metric, which accounts for species presence/absence but not abundance, were distinct from the weighted UniFrac plots, demonstrating differences in the abilities of the five programs to accurately reflect the species composition versus composition plus abundance (Fig. 2B). Filtering out species present at <0.1% abundance noticeably altered the relationship of the programs to each other in the PCoA plots. CLARK-S and QIIME/UCLUST exhibited substantial differences in community structures between ancient and modern data sets. Filtering removed this difference only for CLARK-S, while QIIME/UCLUST modern and ancient data sets remained distinctly plotted, suggesting that QIIME/UCLUST reported several taxa not in the input files at higher abundance than the cutoff of 0.1%. In contrast to the weighted UniFrac PCoA plots, the MetaPhlAn2 community structure was most similar to the actual data set for 40-, 100-, and 200-genome data sets, in filtered and full tables, followed by MIDAS. Filtering of output tables reduced the community structure similarity between MIDAS and the actual data set and made the community structures of CLARK-S and MALT more similar to each other, suggesting that the most abundant species are detected in similar proportions by CLARK-S and MALT. Using the nonphylogenetic Jaccard distance, we observed similar PCoA plotting patterns by each program, relative to the actual input, at the species and genus levels (see Fig. S3 to S5).

### Community diversity is program dependent.

**FIG 3:**
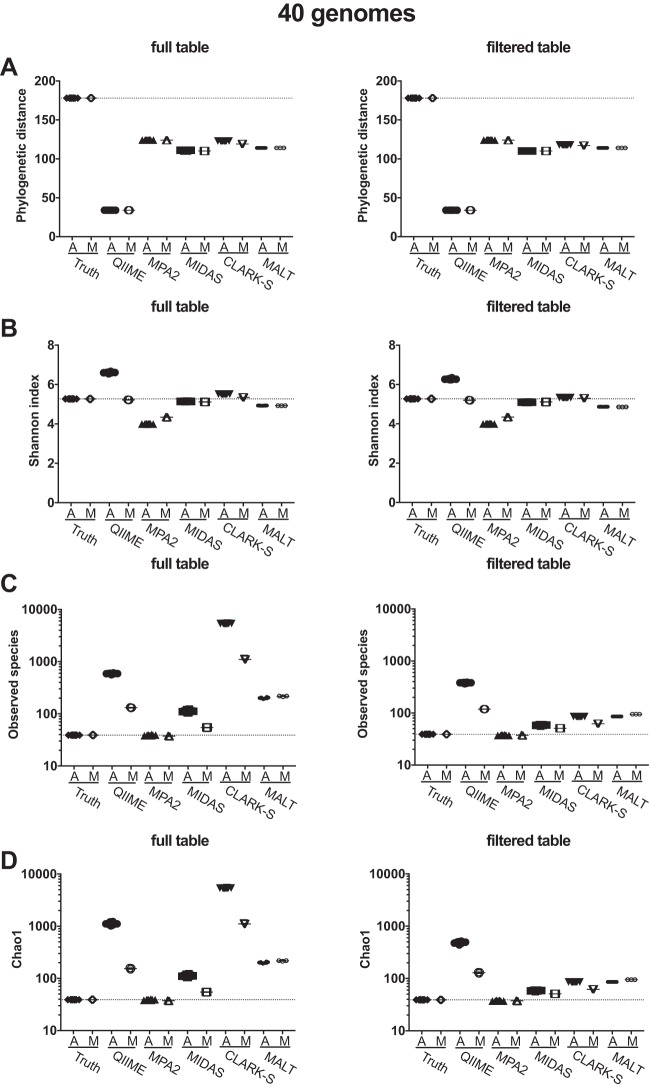
Damage patterns slightly increase within-sample diversity. Alpha diversity of 40-genome data sets was calculated by (A) Faith’s phylogenetic distance, (B) Shannon index, (C) observed species, and (D) Chao1 for full-output tables and tables filtered to remove species present at <0.1% abundance. A, ancient samples; M, modern samples; MPA2, MetaPhlAn2.

To better understand the differences in community structure we observed in beta diversity analyses, we assessed the community diversity (alpha diversity) of the taxonomic profiles produced by the five successfully tested classification programs. We calculated alpha diversity using several metrics to account for different components of community diversity. Faith’s phylogenetic distance (PD), which determines the community diversity based on the phylogenetic relatedness of the species present, was estimated to be much lower than the true PD by all of the programs for the 40-, 100-, and 200-genome data sets, full and filtered tables, and ancient and modern simulations (Fig. 3A; see Fig. S6A and S7A). QIIME/UCLUST generated the lowest PD, while MetaPhlAn2 and CLARK-S were both slightly higher than MIDAS and MALT. CLARK-S was the only program with a slight difference in PD between ancient and modern simulated data sets, but when the table was filtered, the modern and ancient sample diversities were equivalent.

The Shannon index, which accounts for species presence/absence and evenness, showed little difference between ancient and modern simulated data sets per program and was unaffected by filtering (Fig. 3B; Fig. S6B and see S7B). As the number of genomes in the actual input files increased, the Shannon index values for each program decrease relative to the true value. (That is, in the 40-genome set, QIIME/UCLUST, MIDAS, and CLARK-S are above the true value, and in the 200-genome set, all program values are below the true value.) This may have been caused by the fact that the Shannon index of communities with dominant species is expected to be lower than those with even abundance across species, even if the former communities are more species rich.

The observed species is the total number of species/subspecies detected by each program (except QIIME/UCLUST, which included all OTUs because it poorly resolves species-level differences). QIIME/UCLUST, MIDAS, CLARK-S, and MALT always overestimated the total number of species in the samples (by 1- to 150-fold), and the number of estimated species/subspecies in ancient simulated samples was much higher than in modern simulated samples for QIIME/UCLUST and CLARK-S and, to a lesser extent, MIDAS (Fig. 3C; see Fig. S6C and S7C). Filtering reduced the number of observed species by CLARK-S substantially, by MALT and MIDAS slightly, and by QIIME/UCLUST minimally (see Table S4). In contrast to the other programs, MetaPhlAn2 slightly underestimated the total number of species in all of the data sets and is consistently closest to the true number. Chao1 diversity metrics, which include an estimation of undetected species in the sample, exhibited very similar patterns to observed species for all programs (Fig. 3D; see Fig. S6D and S7D).

### Individual program performance and biases.

We next assessed how well each program detected the presence and abundance of species present in both modern and ancient simulated data sets. To do so, we calculated the true and inferred relative abundance of each input genome for each of the five programs and determined the percentage of over- or underassignment (Fig. 3; see Fig. S8 and S9). Given the limited species-level resolution afforded by QIIME/UCLUST, we limited our analysis to genus-level assignments for this program. MetaPhlAn2 does not distinguish between several species (i.e., Streptococcus mitis and Streptococcus oralis) because their marker genes are indistinguishable, and the relative abundance of these in the input files was likewise combined for calculations. Generally, the species detected/not detected are consistent between the ancient and modern simulated data sets, as is the percentage and direction of over- and underestimation. We have additionally presented (see Fig. 5; see Fig. S10 to S19) the relative abundance of each species as bar charts in the input (labeled “If” for “Input fastq/a” and “16f” for “Input 16S rRNA gene-identified read fastq/a”) and output profiles from each program. The first output profile bar (labeled “Id” for “Input species detected”) excludes the false-positive species not in the input files (grouped together as “other” assignments). The second output profile bar (labeled “Ad” for “All species detected”) includes the “other” assignments to visualize how skewed the proportions of input species are by assignments to taxa not in the input. Assessment of each of the programs is included in the program-specific sections below.

### QIIME/UCLUST.

**FIG 4:**
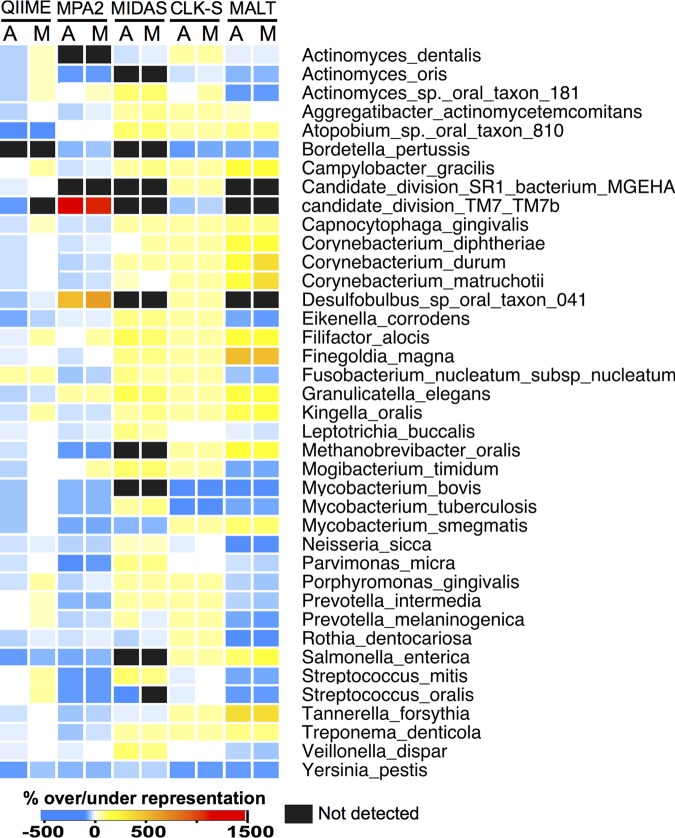
Species detection and over/underrepresentation differ by program but not aDNA damage. The heat map shows for each program tested the species relative abundance underrepresented (blues), overrepresented (yellows, oranges, reds), not detected (black), and accurately represented (white) relative to the true input files for modern and ancient 40-genome data sets. Where programs were unable to distinguish species, strains, or subspecies, a single bar across those genomes is colored to represent the over/underrepresentation of the lowest identifiable taxonomic level. A, ancient samples; M, modern samples; MPA2, MetaPhlAn2; CLK-S, CLARK-S.

**FIG 5:**
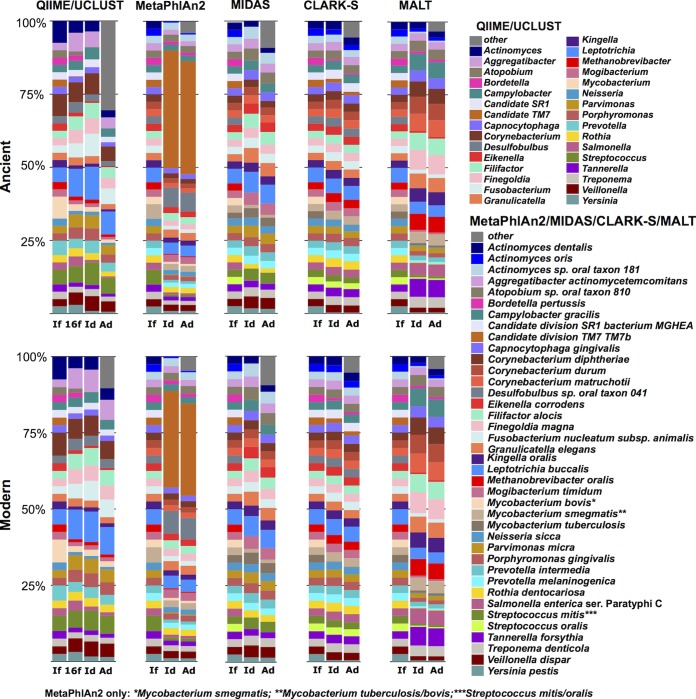
Differences in species relative abundance are program specific and minimally affected by aDNA damage. Program-specific differences in species detection and relative abundance are consistent between ancient (top) and modern (bottom) 40-genome simulated data sets. Relative abundances of each bar are indicated as follows: If, true input fasta file; 16f, 16S rRNA gene input fasta file; Id, input species detected; and Ad, all species detected. Species other than those included in the input files are grouped together as “other” in a gray stripe at the top of the Ad bar. QIIME/UCLUST bars represent genus-level assignments.

QIIME is a popular metagenomics analysis program that was developed to analyze reads generated by 16S rRNA gene amplicon sequencing rather than full metagenome shotgun sequencing data (12). To accommodate this for ancient samples, we used bowtie2 to select the reads from our *in silico* communities that matched 16S rRNA genes in the Greengenes v13.8 database and created new input fastq files containing only those reads, a protocol that has been previously used to enable QIIME analysis of ancient metagenomic sequences (8). The taxonomic proportions of the 16S rRNA gene input files were initially skewed by the bowtie2 identification such that some taxa were overrepresented while others were underrepresented relative to the full genome proportions (see Fig. 5; see Fig. S10 to S11 [bars If versus 16f] and Table S3). Since the 16S rRNA gene does not provide species-level resolution for many species, we assessed the accuracy of assignments at the genus level. QIIME/UCLUST failed to identify 2, 17, and 19 input taxa in the 40-, 100-, and 200-genome simulated data sets (22 total input taxa comprising 16 genera) (Fig. 4; see Fig. S8 and S9 and Table S5), despite the presence of reads derived from these 22 genomes in the bowtie2 16S rRNA gene-identified read files. Of the missing taxa, 11 are not included in the Greengenes v.13.8 database at the species or genus level.

**FIG 6:**
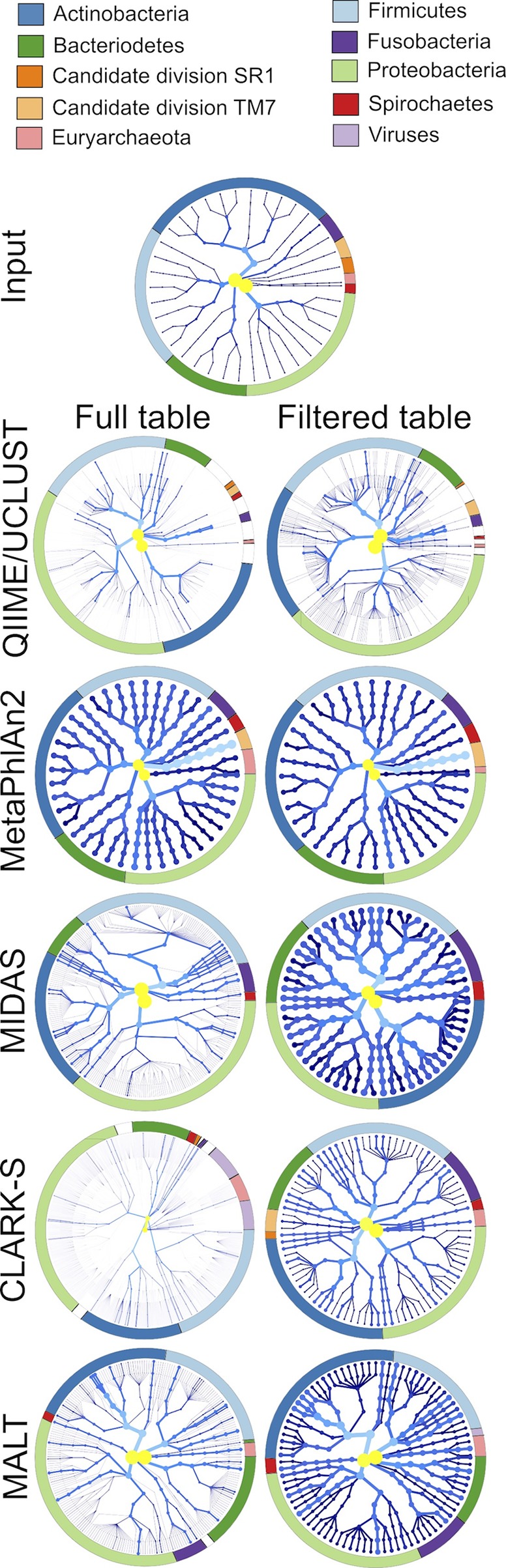
Biases in species detection across the phylogenetic tree are database dependent. The species detected by each program are represented in a radial phylogenetic tree, with the nodes representing different taxonomic levels, where the innermost node is the root and the outermost nodes are strains. More highly represented taxa are lighter in color (yellow to light blue) and have thicker branches/nodes, while less abundant taxa are darker blue with thinner branches/nodes. The ring encircling each tree designates the major phyla (those in the input files, plus viruses when distinguishable) by color. For programs that did not report strains (QIIME/UCLUST, MetaPhlAn2, CLARK-S, and MALT), the species was repeated as a strain to maintain consistency with MIDAS.

QIIME/UCLUST identified the highest proportion of false-positive taxonomic assignments (Fig. 5; see Fig. S10 and S11 [“Other” in the bar chart figure]), and the proportion of false-positive taxa was significantly higher (*P* < 0.01, Fisher’s exact test) (see Table S4) in ancient than modern simulated data sets. This indicates that damage patterns decreased the accuracy of this program to generate accurate taxonomic identifications. Because of the large number of false-positive taxa identified, as well as the several taxa remaining unidentified, many of the input taxa were underrepresented in the OTU tables produced by QIIME (Fig. 4 and 5; see Fig. S8 and S9). Circular trees generated in metacodeR representing the taxonomy of the OTUs identified in the 40-genome ancient data set full and filtered table taxonomic assignments (Fig. 6; see Fig. S20) demonstrated that QIIME/UCLUST tends to overestimate each phylum in proportion to the original input, except for poorly characterized taxa such as Candidate divisions TM7 (“*Candidatus* Saccharibacterium”) and SR1, *Spirochaetes*, and *Archaea*, and there is a slight bias toward overassignment of *Proteobacteria*.

We identified several genus-level false-positive taxa with particularly high assignments in the 40-, 100-, and 200-genome data sets individually, as well as seven that were shared by all three data sets (see Table S5). Three of the seven genera, *Bacteroides*, *Coprococcus*, and *Enterococcus*, had high numbers of assignments only in the ancient simulated data sets, while *Achromobacter*, *Actinobacillus*, *Enterobacter*, and *Erwinia* were highly represented in both ancient and modern simulated data sets. The genomes from which the reads assigned to each of these seven false-positive taxa originated were identified (see Table S4), and we tested if these assignment biases held true in real data sets. All reads assigned to the seven false-positive genera in a set of historic calculus samples from the Radcliffe Infirmary burial ground (ca. 1770 to 1855; Oxford, United Kingdom) (3) were searched against the NCBI nt database using BLASTN to identify the likely species of origin for the reads. Many of the biases in read assignment observed in the *in silico* data sets were also observed in the real calculus samples (see Table S5 in figshare): i.e., *in silico*-generated reads assigned to *Enterococcus* by QIIME were from taxa in the order *Lactobacillales*, and historic calculus reads assigned to *Enterococcus* by QIIME also had best BLAST hits to the order *Lactobacillales*.

### MetaPhlAn2.

MetaPhlAn2 is a fast program that assigns taxonomy based on single-copy marker genes that are unique to each species in the MetaPhlAn2 database (14, 27). It was shown to be highly accurate for assigning taxonomy in modern metagenomic samples (14, 15), and it is implemented in metaBIT (28), a user-friendly wrapper program that is targeted to ancient metagenomics researchers. MetaPhlAn2 identified the smallest number of false-positive taxa of the programs tested (see Table S6) and showed no significant different in false-positive assignments between modern and ancient DNA. MetaPhlAn2, however, had exceptionally skewed proportions of 2 identified taxa (“*Candidatus* Saccharibacterium” TM7b and *Desulfobulbus* sp. oral taxon 041), which may explain why weighted UniFrac distance community structures were so divergent from the actual data set (Fig. 2; see Fig. S3 to S5), while unweighted UniFrac distance community structures were highly similar to truth, where truth represents the percentage of DNA from a genome rather than the cell count. Circular taxonomic assignment trees of the ancient data set demonstrate that MetaPhlan2 does not report high numbers of false-positive taxa in any phylum (Fig. 6; see Fig. S20), and although there are three more *Proteobacteria* reported than in the input files, they were identified at low abundance and removed during filtering. The only species not represented in the MetaPhlAn2 output data set is from the phylum Candidate division SR1, which is not in the MetaPhlAn2 database.

“*Candidatus* Saccharibacterium” TM7b was represented at 1,500 to 2,000% higher relative abundance in the output files than the abundance of DNA in the input files in 40-, 100-, and 200-genome data sets, both ancient and modern (Fig. 4 and 5; see Fig. S8 and S9 and S12 and S13). This may be the result of the MetaPhlAn2 normalization method, which calculates the proportion of cells from each species based on single-copy marker genes, rather than reporting the relative abundance of all DNA from each species detected (14). The TM7 genome is much smaller than the genomes of the other species we included in our data set (0.1 Mb versus 2.5 to 3.5 Mb), and because our data sets possess the same number of reads from each species, there are more copies of the small-genome cells in the data sets to achieve the same proportion of DNA. We calculated that our data sets have approximately 7.8 copies of the TM7 genome but on average 0.36 copies of all other species genomes, which is a difference of 21.6-fold (~2,000%) (see Table S1). *Desulfobulbus* sp. oral taxon 041 was identified at 200 to 300% higher relative abundance in all output files, and *Prevotella* sp. oral taxon 299, present only in the 200-genome data sets, was identified at 200 to 300% higher relative abundance in the output files (Fig. 4 and 5; see Fig. S12 and S13). Both of these organisms have small genomes (~0.7 Mb), and like TM7b, they have more cell copies per data set than the average (1.2 versus 0.36), which is a difference of 3.3-fold (~340%).

Twenty-three input taxa were not specifically identified in any of the simulated data sets, and all were missing from the MetaPhlAn2 database or not present at the appropriate taxonomic level (see Table S6). Nine of the missing 22 taxa are subspecies, including four within Fusobacterium nucleatum, one within Mycobacterium avium, and four within Salmonella enterica subsp. enterica (see Table S6), and these were identified to the species level (or in the case of Salmonella enterica to the subspecies) but not lower. Several species are indistinguishable by the marker genes used by MetaPhlAn2 and are grouped together, including Streptococcus mitis/S. oralis, Bordetella bronchiseptica/B. parapertussis, and Mycobacterium tuberculosis complex (M. tuberculosis/M. bovis/M. canetti/M. africanum). In order to specifically identify any of these species, other programs will need to be employed. The 40-genome data set had seven false-positive taxa, the 100-genome data set had 12, and the 200-genome data set had 14, yet all were low abundance, suggesting that MetaPhlAn2 may be slightly less accurate at making assignments in samples with higher diversity and may minimally inflate that diversity. Only one false-positive taxon, Streptococcus tigurinus, was common to the 40-, 100-, and 200-genome data sets, and this may be because of inconsistencies in naming this *Streptococcus* species, where some NCBI entries use S. tigurinus as an independent species and others use it as a subspecies of S. oralis. The reads assigned to S. tigurinus may be from S. oralis subsp. tigurinis, which was one of the input genomes we used.

### MIDAS.

MIDAS is a fast program that uses a panel of 15 single-copy marker genes present in all of the species included in its database to perform taxonomic classification (16). It also has the ability to determine differences in gene presence/absence and detect single nucleotide polymorphisms (SNPs), although these were not tested in this study. MIDAS makes use of a substantial database (~31,000 genomes) in which related species are grouped together under a single species identifier number (5,952 total identifiers), which we found introduced biases in the species reported in the output tables. A majority of the species detected in each data set were found only in ancient samples (82%, 72%, and 64% in the 40-, 100-, and 200-genome data sets, respectively [see Table S4]), yet these had a relative abundance of <0.1%. The false-positive rate in the aDNA data set was significantly higher than in the modern data set, indicating aDNA damage patterns do lead to false assignments in MIDAS (see Table S4). This signal, however, disappeared when low-abundance taxa were filtered out.

*Streptococcus* species were the most common low-abundance false-positive taxa and likely indicate a database bias. Biases in reporting *Firmicutes* and *Proteobacteria*, and to a lesser extent *Actinobacteria*, in ancient data sets can be seen in the circular taxonomic assignment trees (Fig. 6; see Fig. S20). Filtering of low-abundance hits removes many of the false-positive taxa in these phyla, yet several lower-abundance (darker nodes/branches) false-positive taxa remain in each. MIDAS did not report any *Archaea*, despite having the input species in the database, nor does it detect Candidate divisions TM7 and SR1, which are not in the database.

In total, MIDAS failed to identify 28 input taxa, the highest number missed of all the programs we tested, and only 3 of these missed taxa were not in the database (Fig. 4; see Table S7 and Fig. S8 and S9). Despite not detecting so many species, MIDAS maintained relative proportions of the input taxa in an even species distribution, and the proportion of false-positive taxa detected was slightly lower in ancient simulated data sets than modern sets (Fig. 5; see Fig. S14 and S15). To understand why we observed certain abundant false-positive taxa, we investigated the origins of the reads assigned to five false-positive taxa that were highly abundant in the 40-, 100-, and 200-genome data sets. We determined which, if any, additional species shared the same MIDAS-specific species identifier number and the origins of the reads being assigned to these false-positive taxa (see Table S6). Reads from several taxa not reported by MIDAS were assigned to false-positive taxa, explaining both why certain taxa were missed and the high abundance of the false positives.

This phenomenon highlights how grouping related species under a single species identifier and presenting only one of those species in the output table can result in inaccurate species profiles from MIDAS. For example, Phocaeicola abscessus, which had high abundance in 100- and 200-genome data sets but was not part of the input files, shares an identifier number with *Bacteriodetes* oral taxon 272, which was in the input files but was absent from the final species tables MIDAS produced. By checking the alignment files that MIDAS generates, we determined that the reads from *Bacteriodetes* oral taxon 272 were assigned to the species identifier shared by these two organisms. The same was true for other false-positive taxa/missing pairs of taxa, including *Actinobaculum* sp. and *Actinobaculum* sp. oral taxon 183, Bordetella bronchiseptica and B. pertussis/B. parapertussis, Synergistetes bacterium and Fretibacterium fastidiosum, Fusobacterium nucleatum CC53 and Fusobacterium nucleatum subsp. vincentii, and “*Candidatus* Prevotella” and *Prevotella* oral taxon 317 (see Table S7). Most of these biases are against oral taxa, which is not surprising for a program developed using nonoral microbiome sources. MIDAS also has difficulty making assignments to the genera *Neisseria*, *Fusobacterium*, and *Salmonella* (see Table S7) and slightly overestimates M. tuberculosis and Y. pestis (see Fig. S8 and S9), suggesting a slight bias for potentially human-pathogenic organisms.

### CLARK-S.

CLARK-S, a version of the CLARK sequence classification system (19, 20), uses spaced k-mers to match reads to whole genomes in a database and was developed specifically to classify reads in metagenomic samples. It performs similarly to Kraken (18), makes assignments only at the taxonomic level designated by the user (default species), and cannot report strains or subspecies. As the database size for CLARK-S increases, the amount of memory required to generate and load the hash table increases substantially, and our database of 16,855 genomes required 1 TB of memory (necessitating use of a high-performance computing [HPC] cluster), yet the program classified each sample in a few hours. CLARK-S was the only program that detected all of the species in the input files that were in the database (see Table S8). (All of the genomes used to create the input files were deliberately included in the CLARK-S custom database.) However, CLARK-S also reported the highest number of false-positive taxa (~6,000 in each 40-, 100-, and 200-genome data set) in both modern and ancient data (see Table S4). The false-positive rate, however, was significantly higher in aDNA data sets than in modern data (see Table S4).

A majority of the species detected were present only in ancient simulated data sets (80%, 75%, and 69% of species in 40-, 100-, and 200-genome data sets, respectively), and the overwhelming majority were present at <0.1% abundance (Fig. 1A and B; see Fig. S1A to D and Table S4). As filtering all species with a relative abundance of <0.1% removed most of the low-abundance false-positive taxa but only 1 to 2% of the total assigned reads, we recommend filtering all tables generated by CLARK-S. There was no clear distinction between high- and low-abundance false-positive taxa, unlike in several other programs we tested. Instead there was a steady decrease in the abundance of false-positive taxa, with a long tail of very-low-abundance species (Fig. 1; see Fig. S2).

Circular taxonomic assignment trees of the CLARK-S unfiltered tables show slight biases for *Actinobacteria* but mostly overestimate each phylum in proportion to the original input (Fig. 6; see Fig. S20). A substantial number of viruses were reported, though all were reported at <0.1% abundance and removed by filtering. Most of the input species were detected by CLARK-S at proportions close to those of the input files for 40-, 100-, and 200-genome data sets, both ancient and modern (Fig. 5; see Fig. S16 and S17), but it was poorly able to detect the genera *Bordetella*, *Burkholderia*, *Mycobacterium*, and *Yersinia* (Fig. 4; see Fig. S8 and S9). Generally, species overestimation was lower in the modern than ancient samples, but underestimation was not consistently different between ancient and modern sample sets (Fig. 4; see Fig. S8 and S9).

### MALT.

Like CLARK-S, MALT (21) makes use of spaced hashes to classify reads to the genomes in a database, and it is the only program we tested that can align reads to a protein database, done through BLASTX, which also allows functional characterization of the microbial community. We ran MALT in BLASTX mode (22) to assess how translating the ancient simulated metagenomic reads affected taxonomic profiles, using a database consisting of NCBI RefSeq nonredundant bacterial, viral, archaeal, and plasmid protein sequences (57,435 species/strains). The amount of memory required to load the hash table into memory was >1 TB (again necessitating use of a high-performance computing cluster), and the program classified samples more slowly than CLARK-S, requiring several hours longer per sample than CLARK-S. The output files were uploaded to MEGAN6 (29, 30), and read count and relative abundance tables of only species-level assignments were exported, although MALT/MEGAN6 does place reads higher up on the taxonomic tree if they cannot be assigned to a species with high confidence. Fourteen input taxa were missing from the output files, nine of which were not in the database (see Table S9). However, reads from each of these taxa were assigned to higher taxonomic levels and in low numbers to closely related species that were not in the input files.

MALT overestimated the number of species in all data sets, but the difference in the total number of assignments between ancient and modern data sets was not significant (see Table S4) and much smaller than that of CLARK-S (Fig. 3C; see Fig. S6C and S7C). Circular taxonomic assignment trees show a bias for *Proteobacteria* that remains after filtering (Fig. 6; see Fig. S20). In the ancient simulated data sets, there were 54, 86, and 75 species detected in the 40-, 100-, and 200-genome data sets, respectively, that were not reported in the modern data sets. The over/underestimation of the relative abundance of each input species was consistent between modern and ancient samples (Fig. 4 and 5; see Fig. S8 and S9 and S18 and S19). The 40-, 100-, and 200-genome data sets each had five false-positive taxa present at >0.1% abundance, while two of these false-positive taxa were reported in the all 3 genome data sets. We observed that MALT assigned a low number of reads to a particularly high number of *Neisseria* and *Prevotella* species that were not in the input files. In the 40-, 100-, and 200-genome data sets, MALT identified 32, 19, and 17 false-positive *Neisseria* species, respectively, and 22, 37, and 34 false-positive *Prevotella* species, respectively, although all of these species were present at <0.1% abundance. This may be because the number of species in the database from these genera is higher than for other species in the input files (such as *Actinobacteria* and *Fusobacteria*).

One unusual false-positive taxon that was consistent with MIDAS was Phocaeicola abscessus in the 100- and 200-genome data sets, both ancient and modern, at a relative abundance of 0.4 to 0.9%. The reads assigned to P. abscessus were all from the *Bacteroides* sp. oral taxon 272 genome, and *Bacteroides* sp. oral taxon 272 was identified at approximately 10% lower relative abundance than P. abscessus in all samples. “*Candidatus* Saccharibacterium” oral taxon TM7x had high numbers of reads assigned to it despite not being in the input file, but it was the only “*Candidatus* Saccharibacterium” TM7 species in the database, and the reads assigned to it were from other TM7 genomes included in the input files. MALT classified a very small number of reads per sample to viruses (<50), but the assignments were not to the species level and were not included in the output files we analyzed.

### Profile differences in a real ancient microbiome data set.

**FIG 7:**
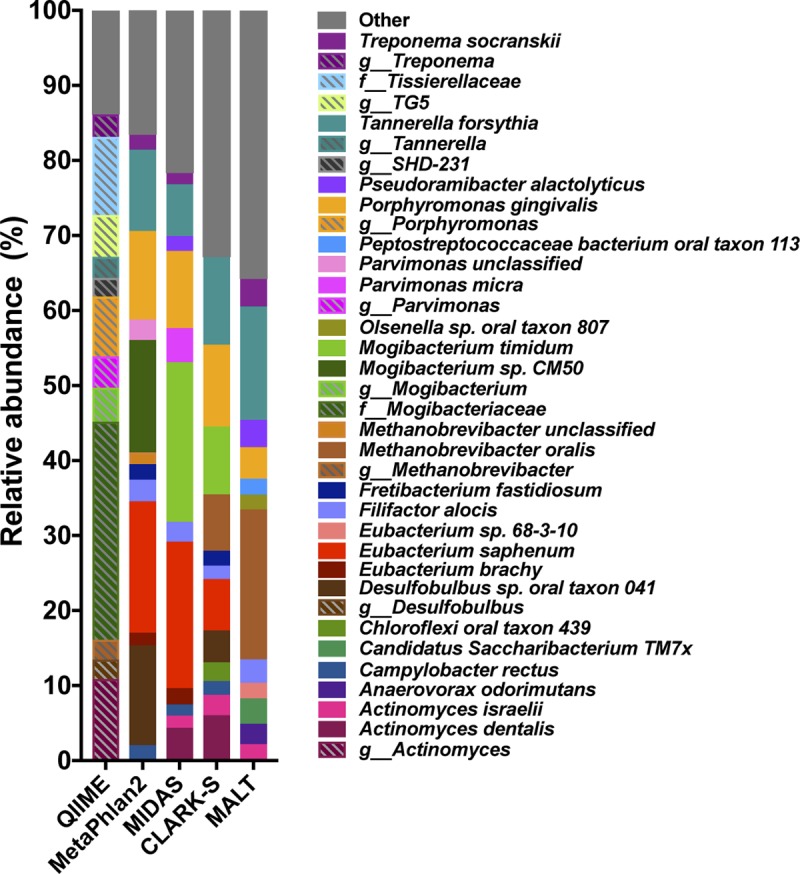
Taxonomic profiles of historic dental calculus differ by classification program. The top 12 most abundant taxa reported by five taxonomic profilers differ substantially in a real historic dental calculus sample (CS32). “Other” includes all species not in the top 12 most abundant.

Finally, we evaluated taxonomic profiles reconstructed by each of the five programs on a real ancient microbiome data set generated from a dental calculus sample (CS32) dating to ca. 1800 to 1850 from the Radcliffe Infirmary burial ground in Oxford, United Kingdom. For consistency, we chose the sample whose damage profile had been used to simulate the damage patterns for our *in silico* experiment. This allowed us to assess how taxonomic profiles differ between programs on a real data set with an equivalent level of preservation. The top 12 most abundant species (OTUs from QIIME) identified by each program are presented in Fig. 7 as bar charts. These species/OTUs consistently accounted for ~65 to 85% of the total microbes reported. Species including Filifactor alocis, Porphyromonas gingivalis, and Tannerella forsythia were reported by all programs at different abundances, while certain genera were reported by multiple profilers, but the specific species differed, including *Methanobrevibacter*, *Mogibacterium*, and *Parvimonas*. In contrast, species including “*Candidatus* Saccharibacterium” oral taxon TM7x, *Chloroflexi* oral taxon 439, and *Olsenella* sp. oral taxon 807 were within the top 12 most abundant taxa of only a single program.

## DISCUSSION

Reconstruction of microbial community composition and structure from short sequencing reads is challenging (31), especially from highly damaged ancient DNA data sets. Here we show that specific programs produce a higher percentage of false-positive assignments in ancient relative to modern data sets, and some are better for comparing microbial profiles between modern and ancient samples. Each program we tested has intrinsic, and at times nonintuitive, assignment biases, and an appreciation of these biases is required to aid interpretation and limit misinformed conclusions.

Targeted amplification of the 16S rRNA gene is popular for profiling complex microbial communities, and programs such as QIIME (12) and Mothur (13) assist with amplicon data processing, binning, and taxonomic profiling based on large 16S rRNA gene databases such as Greengenes (32). DADA2 (33), and Deblur (34) are new methods for binning of 16S rRNA gene reads that have been implemented in QIIME v2.0 and offer more accurate read binning than methods provided in QIIME v1. These programs require multiple copies of each sequence, which although common in 16S rRNA gene amplicon data sets, are not likely to occur in a set of ancient reads that are selected out of a shotgun-sequenced metagenomic data set (such as the ones used in this study) due to insufficient coverage. Targeted amplification of the 16S rRNA gene from highly fragmented ancient DNA samples has been shown to produce strong taxonomic biases due to its long hypervariable regions and length polymorphisms (8), and the 16S rRNA gene is difficult to assemble from metagenomes because of its highly conserved regions (35). As a result, we were unable to bin our 16S rRNA gene-identified reads with DADA2 because each read was represented only once in each of our data sets. We do not recommend using QIIME v2.0 for taxonomic characterization of 16S rRNA gene reads from ancient microbial samples.

Our study does not show that one program clearly outperforms others, but rather each has distinct advantages and disadvantages that are context dependent. For example, for accurate interpretation of community structure, MIDAS is an appropriate choice if measurement of species relative abundance is critical (e.g., by weighted UniFrac distance), while MetaPhlAn2 is more appropriate if this measure is not critical (e.g., by unweighted UniFrac distance). The overall accuracy of MetaPhlan2 and MIDAS suggests that marker genes other than the 16S rRNA gene may be better targets for classification of ancient metagenomes. However, taxonomic accuracy in MIDAS is hampered by the way that the species are reported. For example, MIDAS reduces potential assignments from tens of thousands of genomes in its full database to a more manageable 5,952 ID clusters that are actually used at the taxonomic assignment step. However, it reports as the identified species for each query sequence only one representative species per ID cluster, resulting in inappropriate species profiles despite reads being assigned to an appropriate genome. It may be possible to correct this effect by altering the program to preferentially select a different representative species appropriate for the sample type under analysis, but this would require alteration of the source code or a substantial reanalysis of the output files.

DNA damage differentially affects the false-positive rate of the five programs we tested, but database biases have a much stronger effect on the profiling of the most abundant species. CLARK-S, MetaPhlAn2, MIDAS, and QIIME/UCLUST have greater numbers of false-positive assignments in ancient than modern simulated data sets, although these differences are not significant in MetaPhlAn2. We find that, except for MetaPhlAn2, the majority of false-positive assignments are present at <0.1% abundance. However, for these four programs, the percentage of false-positive assignments is still higher in simulated ancient than modern data sets after filtering the taxa present at <0.1% abundance, and for CLARK-S, this filtering step increases the false-positive rate in ancient relative to modern data sets. This is a concern for studies that wish to compare and contrast microbial profiles of ancient and modern metagenomes, especially for determining the presence and distribution of low-abundance species. Failure to account for a higher false-positive rate in ancient than modern samples may lead to the mistaken conclusion that numerous low-abundance species have been lost through time, when in reality they are false-positive assignments. Similar false-positive rates for ancient and modern data sets are therefore highly desirable, and programs such as MALT and MetaPhlAn2 are more suitable than CLARK-S, MIDAS, and QIIME/UCLUST for these analyses as the ancient and modern sample false-positive rates are comparable both pre- and postfiltering.

One major difference between the different programs lies in the way they compute relative abundance. By using a set of single-copy marker genes, both MetaPhlAn2 and MIDAS attempt to report the proportion of cells of each species detected in a sample. This is in contrast to k-mer-based methods such as CLARK-S and MALT, which report the proportion of total DNA assigned to each species. This difference may explain why the community structures (beta diversity) reported by MetaPhlAn2 and MIDAS were closest to the simulated values. Genome size can vary substantially between bacterial species, and those with larger genomes may appear more abundant in a sample because a higher proportion of DNA is from those species, even though the number of cells may not be higher. Species relative abundance reported by k-mer-based identification methods can be normalized by predicted genome size in order to approximate cell copy number even when the exact strain is not known, as genome size is largely consistent within species. The distinction between the relative abundance reported by cell copy-normalizing (MetaPhlAn2 and MIDAS) and nonnormalizing (Clark-S, MALT, QIIME) metagenomic profilers should be kept in mind when considering metagenomic community profiles.

CLARK-S is best for maximizing the number of assigned reads or determining the relative abundance of all DNA fragments (if, for example, one wants to attempt genome assembly from all reads assigned to a species). Detection of genuine low-abundance species, however (especially viruses and bacteriophages), cannot be achieved with CLARK-S due to a high rate of false-positive identification with abundance lower than 0.1%. MALT is unique in that it can provide functional classification of reads as well as taxonomic classification, but it has difficulty making assignments when the database used has a high number of closely related species (discussed below). In addition, similarly to CLARK-S, MALT has a high rate of false-positive assignment at low abundance. QIIME/UCLUST provides the least accurate method, which included many false positives even when low-abundance taxa were filtered out. In addition, our results indicate that it is the only program whose performance was distinctly different between ancient and modern samples, and the differences could not be resolved by removing low-abundance taxa.

Most of the program-specific biases we observed were due to the database each program used. Familiarity with the taxa present in modern samples is important to ensure appropriate species representation in the database being used and to customize the databases when possible. This will be much more straightforward for relatively well-characterized human body sites such as the mouth (4), and to a lesser extent the gut (36), but will be more nuanced for poorly characterized communities, such as those from nonmodel organisms (37–39). For example, the default RefSeq bacterial database downloaded by CLARK-S does not include any species of *Actinomyces* and has very few species of *Prevotella*, both of which are prevalent and highly abundant oral genera, and the latter of which is major taxon in the gut microbiota of traditional societies (40). Restricting the database to RefSeq genomes alone, such as we did for MALT, limits the genomes to those that have been quality checked and curated, and most sequenced genomes have not met these criteria, nor have metagenome-assembled genomes. Finally, the Greengenes taxonomy has not been updated since 2013 and contains now obsolete taxonomic classification for some organisms, which can confuse results, and more recently updated taxonomic classification systems (41) should be used.

Although ancient dental calculus is highly resistant to taphonomic processes and infiltration of environmental contaminants, it is not immune to them, and paleofeces and other noncalcified archaeological specimens (42) are particularly susceptible to environmental contamination and degradation. Environmental microbes, particularly from soil burial matrix and skin of individuals handling the samples, may remain associated with archaeological samples after cleaning and sterilization and contribute to the metagenomic profile generated by sequencing. Distinguishing environmental signatures from endogenous signatures will be critical for ensuring accurate reconstruction of host-associated microbial profiles. Although outside the scope of this discussion, most microbial databases are heavily dominated by human-associated bacteria, and this may bias the assignment of soil and environmental species. Approaches for limiting false identification of environmental microbial species as host-associated species are discussed by Warinner et al. (11).

The simulated ancient metagenomic data sets we generated were modeled after data generated from archaeological dental calculus (3), and we selected 5 million reads for the *in silico* samples because this was the lowest read count in these samples. However, McIntyre et al. (25) have shown that as read depth increases, the performance of metagenomic classifier tools changes, and this should be kept in mind for studies with higher sequencing depth. We chose not to normalize the output from each program to a consistent taxonomic level, such as genus, because we wanted to work with data that were as close to the default output as possible. This allowed us to see the resolution limit of the programs with respect to known species, subspecies, and strains, as well as the strengths and weaknesses of that resolution. While higher taxonomic classification may demonstrate broad community-level changes, the immense genetic variation in strains of a single bacterial species—for example, Streptococcus mutans (43)—prevents accurate prediction of changes in metabolic functional capacity from higher-order taxonomy.

It is important to note, however, that while community resolution is lost when reads are assigned to higher levels of taxonomy, this technique may ultimately retain more information. Community structure may be better estimated at levels of taxonomy higher than species because reads that do not have species-level resolution can be classified at higher taxonomic levels with greater confidence. Using a lowest-common-ancestor (LCA) algorithm, MALT assigns reads to higher taxonomic levels if they cannot be distinguished between two nearly genetically identical species. For example, some species within the genera *Yersinia* (Y. pestis and Y. pseudotuberculosis) and *Bordetella* (B. pertussis, B. parapertussis, and B. bronchiseptica) are highly genetically similar, and reads that map equally well to multiple species in those genera are usually assigned at the genus level by the LCA algorithm in MALT. Similarly, QIIME/UCLUST will classify reads to deeper nodes in the tree if they cannot be assigned to lower taxonomic levels. For example, the percentages of reads in our data set assigned to different taxonomic levels were as follows: species, 17%; genus, 65%; family, 13%; order, 2.4%; and class, 0.7%. Users should be aware of this behavior in specific programs and be aware of the node at which reads from those taxa tend to assign, since this can substantially affect analyses performed only at the species level.

Assignment of taxonomy to reads below species level is desirable to understand the functional capacity of the microbial community, but the programs we tested performed this task poorly. The ability of MIDAS to discriminate strains or subspecies varies considerably by organism. For example, the 12 strains of Porphyromonas gingivalis in the database share the same species ID, while the 31 strains of Streptococcus mitis each have a unique species ID. This resulted in the MIDAS-produced species profiles containing one strain of P. gingivalis in the 100- and 200-genome data sets (although there are two and four strains, respectively) and 29 to 30 strains of S. mitis across each of the 40-, 100-, and 200-genome data sets (albeit all very low abundance), despite all three data sets containing only one strain. To avoid biases of strain-level identification by this program, we combined all strain-level assignments of the same species into one species-level assignment for all analyses. If identification of subspecies or strains present in a sample is desired, programs specifically designed to perform this function, such as StrainPhlAn (27), Sigma (44), or Platypus Conquistador (45), are recommended instead. Accurate identification of species and strains is particularly important in ancient pathogen studies, and special care should be taken to ensure results are not false positives or derived from modern environmental contamination by following guidelines suggested by Warinner et al. (11) and Key et al. (46).

High proportions of the fecal-associated genera *Coprococcus*, *Enterococcus*, and *Enterobacter* were identified by QIIME/UCLUST in our *in silico*-generated data set, but they were not in the input files. Rather, a high number of reads of consistent taxonomy were assigned to these genera, which we confirmed occurs in real data sets, indicating that these assignments are more likely an artifact of the taxonomic classification process than an indication of poor hygiene. This demonstrates how interpretation of taxonomic assignment results without understanding the biases and limitations of the program used could lead to erroneous conclusions about microbial community profiles and ultimately human activity.

Identification of bacteriophages in ancient metagenomic samples is challenging, and new methods are needed. MetaPhlAn2, CLARK-S, and MALT all detected phages in very low abundance, below levels of suggested filtering to remove spurious assignments. Active bacteriophage replication in the oral biofilm is associated with altered host health status (5, 47), and monitoring of phage activity may offer insight into biofilm pathogenicity in oral (47, 48) and gut (49) sites. Therefore, reliable detection of bacteriophage in host-associated ancient metagenomic samples may allow us to study phage-mediated biofilm changes and evolution relating to human disease. While it is unlikely that we will be able to determine if phage-identified sequencing reads are from viral particles or host-integrated prophages, proteomic characterization of ancient microbiomes (1) may be able to detect viral proteins indicating free phage particles.

Recently, McIntyre et al. (25) assessed the performance of a wide selection of metagenomic taxonomic classification programs built upon a variety of techniques. They reported that the precision of taxonomic assignment can be improved by combining results of certain programs that use different assignment methods, including MetaPhlAn2 and CLARK-S. Combining the results of these taxonomic assignment programs for ancient metagenomics samples may then increase reliability and confidence in historic community structure and composition and should be examined further with *in silico*-generated data sets. Confirmation of species presence/absence by detection with two independent taxonomic classifiers will assist with ensuring specific program biases are not reported as true results.

Analysis and interpretation of real ancient metagenome samples present additional challenges beyond those encountered by analyzing simulated data sets with known composition. In analyzing metagenomic data produced from a real historic dental calculus sample, we observed taxonomic variation with respect to both the presence and abundance of the top 12 most abundant taxa identified by the five profilers. When profiling many samples, these differences can contribute to different outcomes in statistical analyses, such as differential abundance. Using several profiles and running several statistical tests may be warranted to determine if observed patterns are consistent and well supported. Further, mapping reads to one or more representative genomes of a species of interest, such as one with strong differential abundance, may be warranted to confirm its presence.

There are several factors that we did not test that may influence taxonomic profiling of ancient DNA. These include environmental contamination (11) (discussed above), sample location- and age-specific differences in damage patterns (50), and species-specific differential preservation of bacterial DNA (8). Additional *in silico* data set testing, such as by using DNA damage (mapDamage) profiles modeled after older archaeological samples and samples from different locations or based on reads mapped to different or multiple species, may be warranted to determine if and how strongly these factors affect taxonomic profiling. Based on our results that aDNA damage patterns minimally affect read taxonomic assignment, however, we do not expect these variables to substantially alter taxonomic profiles. Nevertheless, location- and age-related biases should be considered in studies that compare samples across geographic locations and/or time.

We have demonstrated that the damage patterns characteristic of ancient DNA do not substantially affect the dominant taxa identified by the five programs that were successfully applied to our data sets. Damage primarily increases the false-positive rate of low-abundance taxa, while variation in the reporting of high-abundance taxa is primarily driven by biases inherent in the programs themselves and the databases they use. This is promising for comparing ancient microbiome samples with modern samples when using the same taxonomic classifier, as biases will be shared by both. Our results highlight the importance of knowing the limitations of the metagenomic classifier being used and investigating any unusual results, such as the presence of unexpected taxa and the absence of expected taxa, to ensure appropriate interpretation of taxonomic profiles.

## MATERIALS AND METHODS

### Simulated ancient and modern metagenomics samples.

Simulated ancient and modern metagenomics fastq files were generated with gargammel (26). Samples of 5 million reads, 99% bacterial and 1% human, were generated with 40, 100, or 200 genomes, with even genome distribution (equivalent numbers of reads from each input genome) and both with and without simulated ancient DNA damage patterns, and sequencing errors were based on Illumina HiSeq2500 150-bp paired-end chemistry and default Illumina adapters. Thirty-nine total metagenomes were simulated as follows: 40-genome even distribution of ancient (10) and modern (3), 100-genome even distribution of ancient (10) and modern (3), and 200-genome even distribution of ancient (10) and modern (3). Genomes are listed in Table S1 and were selected to resemble dental plaque bacterial communities based on the species listed in the Human Oral Microbiome Database (www.homd.org), and relative abundance was roughly based on dental plaque-derived biofilm composition. Select nonoral bacterial species were added to assess biases in detecting specific “pathogenic” species. Although the genomes are represented with equal proportions of DNA in each data set, the number of cells from each organism is unevenly distributed because of differences in genome size (see Table S1).

Ancient DNA damage patterns were simulated based on the mapDamage (9, 10) base composition file and misincorporation file generated on analysis of a real historic dental calculus metagenomic sample (CS32) sequenced on an Illumina HiSeq2500 with 150-bp paired-end chemistry, with bacterial genome damage patterns based on reads mapped to the Tannerella forsythia 92A2 genome (assembly GCA_000238215.1) (see Fig. S1) and human genome damage patterns based on reads mapped to the human genome (assembly GCA_000001405.26) (see Fig. S1), while the fragment length distribution was based on all reads in sample CS21. Simulations for modern metagenomics samples did not include damage pattern input files. The command to simulate ancient metagenomic samples was ./gargammel.pl --comp 0.99,0,0.01 -n 5000000 --misince dnacompCS32e.txt --misincb dnacompCS21b.txt -f fragmentlengthCS21.txt -mapdamagee misincorporationCS32.txt single -mapdamageb misincorporationCS21.txt single -rl 150 -ss HS25 -o output/anc40e1 input/. The command to simulate modern metagenomic samples was ./gargammel.pl --comp 0.99,0,0.01 -n 5000000 -l 150 -rl 150 -ss HS25 -o output/mod40e1 input/. Damage profiles for human (CS21) and bacterial (CS32) reads came from different calculus samples because these had the highest number of reads to the human and T. forsythia genomes, respectively, which allows the most accurate assessment of damage profiles (11). Fragment length distribution for ancient simulated samples was based on calculus sample CS21, while a read length of 150 bp was specified for modern samples. The genome of origin for each read is included in the read name by a gargammel-generated code (listed in Table S1), and the exact number of reads derived from each genome was determined by counting in each of the 78 input fastq files.

### Read processing and 16S rRNA gene fragment filtering.

Reads were processed following a custom pipeline modified for ancient DNA metagenomics samples. AdapterRemoval (51) was used to detect and remove consensus adapter sequences, quality-trim reads at *Q*30 and collapse paired reads. Singleton files were discarded, and reads with residual adapters were detected with bowtie2 (52) and filtered from the samples with filter_fasta.py in QIIME v1.9 (12). Four final files were generated: collapsed reads, pair1 reads, pair2 reads, and truncated collapsed reads, and all 4 files were concatenated to generate a single input file for taxonomic classification. Reads mapping to the 16S rRNA gene were filtered from the cleaned simulated shotgun-sequenced samples and collected in separate files for classification as follows. A bowtie2 database was generated from the Greengenes v13.8 database (32), and the cleaned and collapsed, pair1, pair2, and collapsed truncated fastq files were searched against this database with bowtie2. All reads that mapped to 16S rRNA gene reads were filtered from the full data set fastq files to a separate file using seqtk (https://github.com/lh3/seqtk). All 4 files matching the 16S rRNA gene (collapsed, pair1, pair2, and collapsed truncated) were concatenated for taxonomic classification (see Table S3). Reads mapping to the 16S rRNA gene were filtered from the simulated shotgun data rather than generated independently as amplicons because 16S rRNA gene amplicon sequencing of ancient microbial DNA is known to produce strong taxonomic biases (8) that are not observed in shotgun-sequenced data sets.

### Taxonomic classification.

Reads in all simulated metagenomics samples were classified with 5 taxonomic identification programs (see Table S2): QIIME v1.9/UCLUST/Greengenes v13.8 database (12, 32, 53), MetaPhlAn2 (14, 15), MIDAS (16), CLARK-S (20), and MALT (21) run in BLAST-X mode (22). Input for all programs except QIIME were the full set of cleaned reads, while for QIIME it was the full set of cleaned 16S rRNA gene-identified reads. All options that differed from default are listed in Table S2. Each program uses a different classification method. QIIME v1.9 was used to bin reads matching the 16S rRNA gene using UCLUST (53) with pick_closed_reference_otus.py and to assign taxonomic classification with the Greengenes v13.8 database at 97% identity (202,421 sequences, 99,322 OTUs). We used a customized workflow in QIIME with OTU-picking parameters that had been modified for shotgun-sequenced metagenomic ancient DNA (see Table S2); 16S rRNA gene amplicon sequencing was not attempted because it is known to produce biased taxonomic profiles from ancient samples (8). Closed-reference OTU picking rather than open-reference OTU picking was performed because this is necessary for metagenomic data in which reads consist of nonoverlapping segments of the 16S rRNA gene. The Greengenes database was chosen because it is a curated database, with fewer misannotated sequences than other 16S rRNA gene databases, despite not being updated for several years.

Samples were not rarefied to identical OTU counts prior to analysis, as this practice is not recommended (54), and the standard deviation in the number of classified reads between samples in each data set was less than 100. The output biom file was summarized at the species level, which included all assignment levels from kingdom through species. MetaPhlAn2 and MIDAS used their respective default databases (16,904 species/strains and 31,007 genomes/5,952 species groups, respectively), while CLARK-S was run against a custom database of 16,855 genomes, and MALT was run in BLAST-X mode against a custom database of NCBI RefSeq nonredundant bacteria, viral, archaeal, and plasmid protein sequences (57,435 species/strains). MetaPhlan2 and CLARK-S output were set to species level. The MALT output rma6 files were uploaded to MEGAN6 (30), and classification tables of species assignments only were exported. Output for each classification program is unique, with MetaPhlAn2 and MIDAS providing relative abundance on a scale of 0 to 100 and 0 to 1, respectfully, QIIME and CLARK-S providing a read count table, and MALT providing both relative abundance and read counts.

Outputs were normalized in 2 ways, generating 2 sets of tables: relative abundance of all assignments on a scale of 0 to 100 was calculated based on the number of reads assigned, if provided (QIIME, Clark-S, MALT), and pseudo-read counts were determined by multiplying the relative abundance by the total number of reads in the input files for MetaPhlan2 and MIDAS. The true input tables were also converted to biom format in count read and relative abundance formats. The NCBI taxonomy ID of each taxonomic assignment in each program was determined and used to create a single taxonomy file to assign taxonomy to biom files. All output tables, read counts, and relative abundances were converted to biom format in QIIME v1.9, and taxonomy based on NCBI taxonomy ID was added to each. To determine if removal of very-low-abundance assignments improved the profiles, a second set of biom files was generated by removing all assignments present at less than 0.1% abundance (filtered tables). All biom files were summarized at the phylum, class, and genus levels in QIIME v1.9, using summarize_taxa.py, to allow assessment of classification biases at different taxonomic levels. Mapping data, including the simulated age of the sample (ancient or modern) and the taxonomic assignment program, were added to the biom files in QIIME v1.9.

QIIME v1 is no longer being supported with the release of QIIME v2.0, and QIIME v2.0 uses different taxonomic assignment programs from QIIME v1: DADA2 (33) and Deblur (34). We also tried to include DADA2 in this assessment (see Table S2), using 16S rRNA gene-identified reads and the DADA2 R package as follows. AdapterRemoval was run on the simulated samples as before, but pair1 and pair2 reads were not collapsed. The reads matching 16S rRNA genes were identified using bowtie2 and the Greengenes v13.8 database as before and filtered out of the pair1 and pair2 files. The pair1 and pair2 files of 16S rRNA gene-identified reads were used as input in DADA2. DADA2 was not able to merge sequences in any file because all were unique, and this prevented DADA2 from performing the sequence variant calling, and the program was unable to perform taxonomic assignment. Therefore, we were unable to proceed with DADA2, and have no results to present.

We tested whether the number of false assignments was higher in simulated aDNA libraries than in simulated modern DNA libraries by Fisher’s exact test using R on both unfiltered and filtered taxonomic assignment tables. Three of the 10 ancient simulated data sets were randomly subsampled for unbiased comparison with the three modern simulated samples.

### Diversity metrics.

Alpha diversity was calculated in QIIME using the metrics Faith’s phylogenetic distance, Shannon index, observed species, and Chao1, using count read and pseudocount read files, and graphs were generated using Prism v7. Beta diversity was calculated on relative abundance biom files and plotted using the R package phyloseq (55) for the metrics UniFrac (56) (which accounts for phylogenetic relatedness and presence/absence) and weighted UniFrac (57) (which accounts for phylogenetic relatedness, presence/absence and abundance), Bray-Curtis (which accounts for presence/absence and abundance), and binary Jaccard (which accounts only for presence/absence). A Newick-formatted phylogenetic tree was generated with phyloT (http://phylot.biobyte.de), including the NCBI taxonomy IDs of all assignments made by each program (9,919 total IDs), using the Internal nodes-Expanded and polytomy-Yes options.

### Program assignment biases.

All output text files were manually inspected for taxonomic assignment biases. When a species in the input files was not detected by a program, the database of that program was searched for that species to understand why it was missed. The percentage of over/underrepresentation of each genome compared to the input file was calculated (relative abundance in output/relative abundance in input × 100) and plotted as a heat map with the R library gplots (http://www.rdocumentation.org/packages/gplots). The percentage of each species in the input files detected by each program as well as the percentage of all other species detected but not in the input file was plotted in R using ggplot2 (ggplot2.org). The R package metacodeR (58) was used to visualize phylogenetic tree assignment biases in the ancient data sets by each program. Each node is a taxonomic assignment starting with the root (yellow circle), then kingdoms, phyla, etc., radiating off to subspecies level at the tips. For programs that did not produce subspecies- or strain-level taxonomic assignments, the species assignment was repeated, to maintain visual consistency between all trees. The input data for these trees is the species/subspecies level for all programs except QIIME/UCLUST (which included all levels), so the internal nodes sum the leaves moving from subspecies back toward the root. The colors and weight of nodes and branches represent the relative abundance of each taxonomic assignment, where lighter colors with thicker branches are more abundant (yellows and light blues) and darker, thinner branches are less abundant. The relative abundance is the average of all 10 output files for each program. A ring circling each tree and color coding each phylum was added in Inkscape.

We tested whether a QIIME/UCLUST false-positive taxon read assignment bias was present in real ancient metagenomic samples by processing metagenomic data generated from 19th century dental calculus samples (3) through the same 16S rRNA gene selection and QIIME/UCLUST OTU picking and then filtering out all reads assigned to the designated “false-positive” genera. These reads were searched against the NCBI nt database with BLAST using default parameters, and the BLAST hits of the reads assigned to each “false-positive” genus were determined using MEGAN6 and compared to the origin genomes of the false-positive taxon assigned reads from the simulated samples.

### Data availability.

All supplemental figures are available for download on figshare (as a single pdf) at https://doi.org/10.6084/m9.figshare.6203126. All supplemental tables are available for download on figshare (separate tabs in a single Excel spreadsheet) at https://doi.org/10.6084/m9.figshare.5938849. All gargammel-generated “raw” sequencing read files (forward and reverse) are available for download on Edmond at https://edmond.mpdl.mpg.de/imeji/collection/uLUVi8X7gziZGz5H. Cleaned reads for the real historic dental calculus sample (CS32) are available from the NCBI Short Read Archive under accession no. SRR7083204.
